# Synthesis, crystal structure and properties of bis­(iso­seleno­cyanato-κ*N*)tetra­kis­(pyridine-κ*N*)nickel(II)

**DOI:** 10.1107/S2056989023000245

**Published:** 2023-01-12

**Authors:** Christian Näther, Jan Boeckmann

**Affiliations:** a Institut für Anorganische Chemie, Universität Kiel, Max-Eyth-Str. 2, 24118 Kiel, Germany; University of Aberdeen, United Kingdom

**Keywords:** crystal structure, nickel seleno­cyanate, discrete complex, thermal properties

## Abstract

The reaction of nickel chloride hexa­hydrate with potassium seleno­cyanate and pyridine in water leads to the formation of crystals of the title complex, [Ni(NCSe)_2_(C_5_H_5_N)_4_], which were characterized by single-crystal X-ray diffraction. Its crystal structure consists of discrete complexes, located on centers of inversion, in which the Ni cations are sixfold coordinated by two terminal N-bonded seleno­cyanate anions and four pyridine ligands within a slightly distorted octa­hedral coordination. In the crystal, the complexes are connected by weak C—H⋯Se inter­actions. PXRD investigations revealed that a pure crystalline phase has formed. In the IR and Raman spectra, the C—N stretching vibrations are observed at 2083 and 2079 cm^−1^, respectively, in agreement with the presence of only terminally bonded anionic ligands. Upon heating, one well-resolved mass loss is observed, in which two of the four pyridine ligands are removed, leading to a compound with the composition Ni(NCSe)_2_(C_5_H_5_N)_2_. In this compound, the C—N stretching vibration is shifted to 2108 cm^−1^ (Raman) and 2115 cm^−1^ (IR), indicating the presence of μ-1,3-bridging anionic ligands. In its PXRD pattern, very broad reflections are observed, indicating for poor crystallinity and/or very small particle size. This crystalline phase is not isotypic to its Co and Fe analogs.

## Abstract 2

In the crystal structure of the title compound, discrete centrosymmetric complexes are observed, in which the Ni cations are octa­hedrally coordinated by two terminal N-bonded seleno­cyanate anions and four pyridine coligands.

## 1. Chemical context

Coordination compounds based on thio­canate anions are well investigated, which can partly be traced back to their versatile magnetic behavior, including anti­ferro- or ferromagnetic ordering as well as single-chain magnet behavior (Shurdha *et al.*, 2013 ▸; Prananto *et al.*, 2017 ▸; Mautner *et al.*, 2018 ▸; Werner *et al.*, 2014 ▸; Rams *et al.*, 2020 ▸). In contrast, much less is known about the corresponding seleno­cyanate coordination compounds, which might be related to the fact that their synthesis is more difficult to achieve. This is especially the case if less chalcophilic metal cations are used and compounds with bridging anionic ligands are to be prepared. Therefore, only a very limited number of such compounds have been reported in the literature (Turpeinen, 1977 ▸; Vicente *et al.*, 1993 ▸; Wöhlert *et al.*, 2012 ▸). To overcome this problem, we developed a synthetic procedure that allows a more directed preparation of thio- and seleno­cyanate coordination compounds with bridging anionic ligands, which is based on thermal treatment of suitable precursor compounds in which the anionic ligands are only terminally bonded (Werner *et al.*, 2015 ▸; Wriedt & Näther, 2010 ▸). Upon heating, the neutral coligands are usually stepwise removed, leading to the formation of the desired compounds with a bridging coordination as inter­mediates. This procedure works perfectly for the synthesis of thio­cyanates but can also be used for the synthesis of seleno­cyanates (Wöhlert *et al.*, 2012 ▸). 
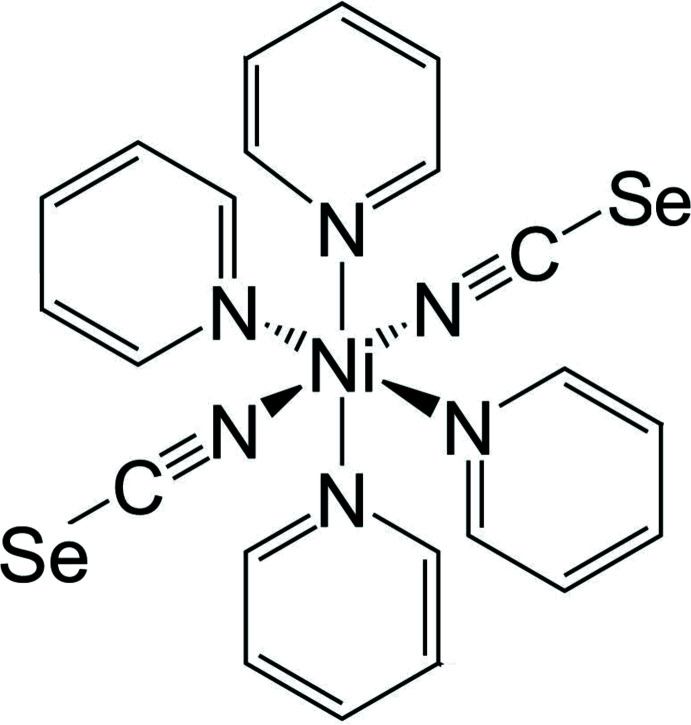


**Figure 1:**
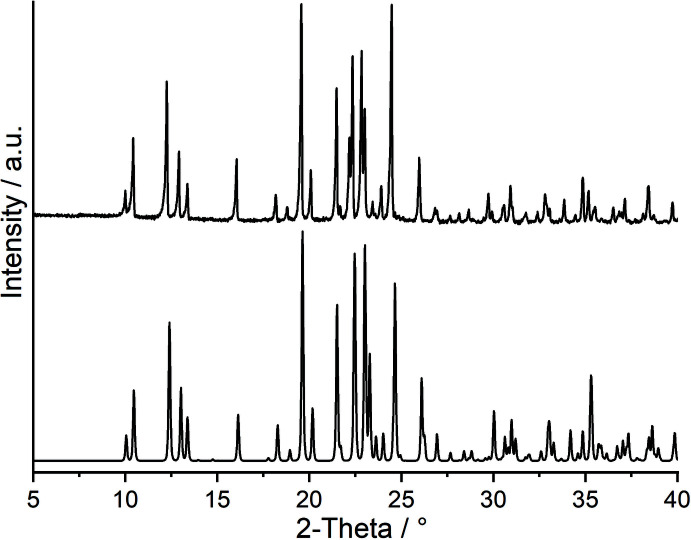
Experimental (top) and calculated PXRD pattern (bottom) of the title compound.

**Figure 2:**
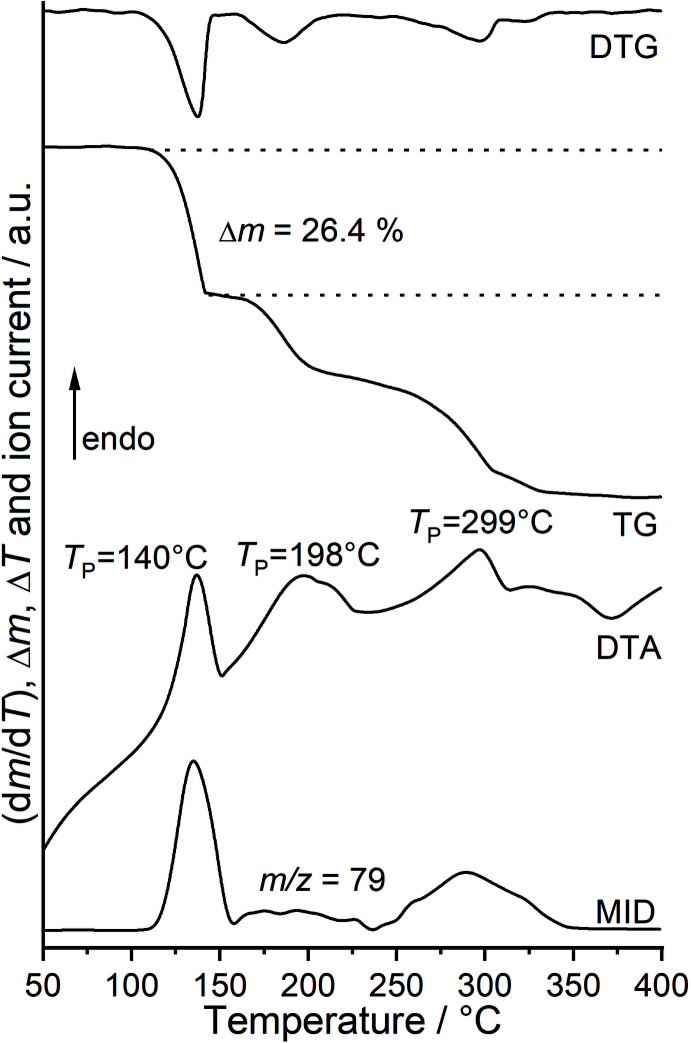
DTG, TG, DTA and MS trend scan curves for the title compound measured at 4°C min^−1^ in helium.

In this context we have reported on compounds with the composition *M*(NCSe)_2_(pyridine)_4_ (*M* = Fe, Co) that upon heating lose two of the pyridine coligands and transform into compounds with the composition *M*(NCSe)_2_(pyridine)_2_ (*M* = Fe, Co), in which the metal cations are linked by pairs of μ-1,3-bridging seleno­cyanate anions into chains (Boeckmann *et al.*, 2012 ▸; Boeckmann & Näther, 2011 ▸). In the course of our systematic work we also became inter­ested in the corres­ponding Ni compounds, which are not reported in the literature. The synthesis of the desired compound Ni(NCSe)_2_(pyridine)_2_ in solution was unsuccessful but we found that single crystals, as well as larger amounts of a microcrystalline powder with the composition Ni(NCSe)_2_(pyridine)_4_, can easily be prepared from solution. The CN stretching vibrations of the anionic ligand are observed at 2083 cm^−1^ in the IR and at 2079 cm^−1^ in the Raman spectrum, which indicates that the seleno­cyanate anions are only terminally bonded (Fig. S1 in the supporting information). A comparison of the experimental powder X-ray pattern with that calculated from single-crystal data reveals that a pure crystalline phase has formed (Fig. 1 ▸). Measurements using differential thermal analysis and thermogravimetry coupled to mass spectrometry (DTA–TG–MS) show one well-resolved mass loss in which the pyridine ligands are emitted and that is accompanied with an endothermic event in the DTA curve at 140°C (Fig. 2 ▸). Upon further heating, the TG curve is poorly resolved and two additionally endothermic events are observed. The experimental mass loss of 26.4% in the first step is close to that calculated for the removal of half of the pyridine ligands (27.0%). Therefore, it can be assumed that in the first mass loss a compound with the composition Ni(NCS)_2_(pyridine)_2_ is formed that, upon further heating, loses the remaining pyridine ligands and that this event cannot be separated from the decomposition of nickel seleno­cyanate at higher temperatures. For this residue, IR and Raman spectroscopy show that the CN stretching vibrations are located at 2115 cm^−1^ in the IR and at 2108 cm^−1^ in the Raman spectrum, indicating that μ-1,3-bridging seleno­cyanate anions are present (Fig. S2). PXRD investigations proved that the reflections of the precursor compound are absent but that a residue of poor crystallinity and/or very small particle size is obtained (Fig. S3). A comparison of the experimental powder pattern with that calculated for Co(NCSe)_2_(pyridine)_2_ retrieved from literature shows that these compounds are not isotypic (Fig. S3). Indexing of this powder pattern failed.

## 2. Structural commentary

**Figure 3:**
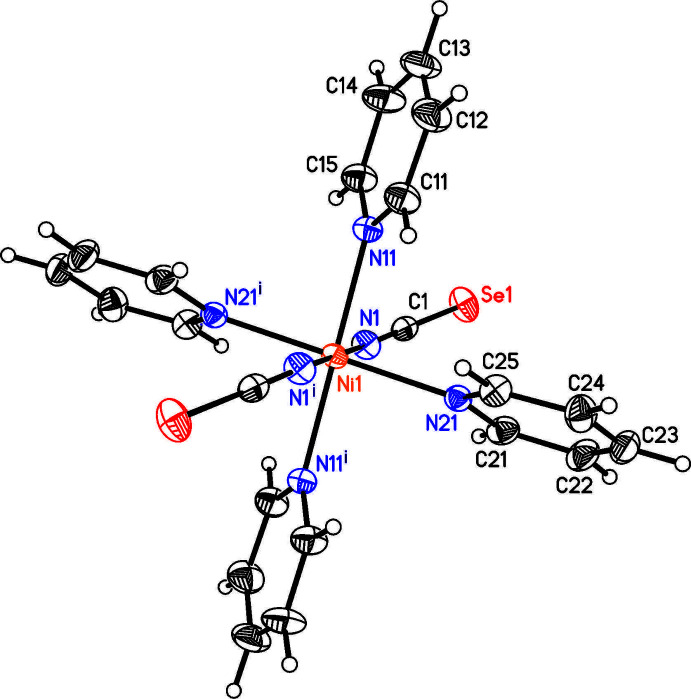
Crystal structure of the title compound with labeling and displacement ellipsoids drawn at the 50% probability level. Symmetry code: (i) −*x* + 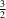 , −*y* + 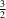 , −*z* + 1.

Single-crystal structure determination proves that the title compound, Ni(NCSe)_2_(pyridine)_4_, is isotypic to its Co, Fe, Cd and Zn analogs already described in the literature (Boeckmann & Näther, 2011 ▸; Boeckmann *et al.*, 2011 ▸ and 2012 ▸). The asymmetric unit consists of one crystallographically independent Ni^II^ cation that is located on a center of inversion as well as one seleno­cyanate anion and two pyridine ligands in a general position (Fig. 3 ▸). The Ni cations are sixfold coordinated by four pyridine coligands and two terminally N-bonded seleno­cyanate anions in *trans*-positions. Bond lengths are similar to those in the corresponding Fe and Co compounds, even if the Ni—N bond lengths are slightly shortened because of the lower ionic radii. From the bond lengths and angles (Table 1 ▸) it is obvious that the octa­hedra are slightly distorted.

## 3. Supra­molecular features

**Figure 4:**
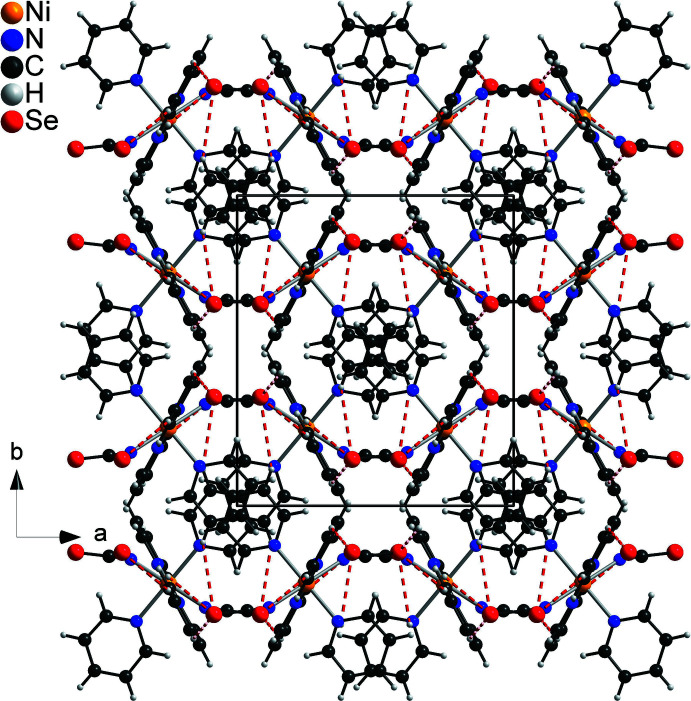
Crystal structure of the title compound with view along the crystallographic *c-*axis direction. C—H⋯Se inter­actions are shown as red dashed lines.

In the crystal, the Ni(NCSe)_2_ units are arranged in corrugated layers in the *ac* plane and the pyridine rings are arranged in columns that proceed along the crystallographic *c*-axis direction with no sign of π–π inter­actions (Fig. 4 ▸). There are some C—H⋯Se contacts, with angles above 150°, indicating weak hydrogen-bonding inter­actions (Table 2 ▸). There are additional C—H⋯N contacts, but distances and especially angles indicate that they should not correspond to any significant inter­actions (Table 2 ▸).

## 4. Database survey

Some seleno­cyanate compounds with pyridine as ligand have been deposited in the Cambridge Structural Database [ConQuest Version 2022.2.0, CSD Version 5.43 (March 2022); Groom *et al.*, 2016 ▸], including isotypic compounds with composition *M*(NCSe)_2_(pyridine)_4_ (*M* = Co, Fe, Cd, Zn) in which the metal cations are octa­hedrally coordinated by two terminal N-bonded seleno­cyanate anions and four pyridine ligands (refcodes ITISOU, CAQVEX, OWOJAM and OWOHUE; Boeckmann & Näther, 2011 ▸; Boeckmann *et al.*, 2012 ▸, 2011 ▸). For these compounds, mixed crystals with the composition Co(NCS)_x_(NCSe)_2–x_(pyridine)_4_ have also been reported (refcodes TIXDOW and TIXDOW01; Neumann *et al.*, 2019 ▸).

There are compounds with the composition *M*(NCSe)_2_(pyridine)_2_ (*M* = Co, Fe, Cd) in which the metal cations are octa­hedrally coordinated by two terminal N- and S-bonded seleno­cyanate anions and two pyridine ligands and are linked by pairs of seleno­cyanate anions into chains (refcodes: ITISUA, CAQVIB and OWOHOY; Boeckmann & Näther, 2011 ▸; Boeckmann *et al.*, 2012 ▸, 2011 ▸). These compounds are also isotypic. There is an additional compound of composition Zn(NCSe)_2_(pyridine)_2_ that consists of discrete complexes in which the Zn cations are tetra­hedrally coordinated by two terminal N-bonded seleno­cyanate anions and two pyridine ligands (refcode OWOJEQ; Boeckmann *et al.*, 2011 ▸).

One mixed-metal compound with the composition HgSr(NCSe)_4_(pyridine)_6_ is also reported, in which the Hg cations are tetra­hedrally coordinated by four Se-bonded seleno­cyanate anions and linked to the Sr cations that are octa­hedrally coordinated by two N-bonded seleno­cyanate anions and four pyridine ligands (refcode CICLOP; Brodersen *et al.*, 1984 ▸).

A dinuclear complex with the composition (Fe(NCS)_2_)_2_(pyridine)_2_((3,5-bis­(pyridin-2-yl)pyrazol­yl)_2_ is found that shows spin-crossover behavior (refcode FIZYEU; Sy *et al.*, 2014 ▸). Finally, there is another spin-crossover complex with the composition Fe(NCSe)_2_(pyridine)_2_-2-methyl­dipyrido[3,2-*f*:2′,3′-*h*)(quinoxaline) pyridine solvate (refcode TISWOI; Tao *et al.*, 2007 ▸).

## 5. Synthesis and crystallization

NiCl_2_·6H_2_O and K(SeCN)_2_ were purchased from Merck and pyridine was purchased from Alfa Aesar.

**Synthesis:**

Larger amounts of a microcrystalline powder were obtained by the reaction of 59.4 mg of NiCl_2_·6H_2_O (0.25 mmol) and 72.0 mg (0.5 mmol) of KSeCN in a mixture of 1.5 ml of pyridine and 1.5 ml of water by stirring for 3 d at room temperature. The precipitate was filtered off and washed with a very small amount of water. Single crystals in the form of purple blocks were obtained under the same conditions but without stirring.

**Experimental details:**

Differential thermal analysis and thermogravimetric (DTA–TG–MS) measurements were performed in a dynamic helium atmosphere in Al_2_O_3_ crucibles using a Netzsch thermobalance with skimmer coupling and a Balzer Quadrupol MS. The XRPD measurements were performed by using a Stoe Transmission Powder Diffraction System (STADI P) equipped with a linear, position-sensitive MYTHEN detector from Stoe & Cie with Cu *K*α radiation. The IR data were measured using a Bruker Alpha-P ATR-IR spectrometer and the Raman spectra were measured with a Bruker Vertex 70 spectrometer.

## 6. Refinement

Crystal data, data collection and structure refinement details are summarized in Table 3 ▸. Hydrogen atoms were positioned with idealized geometry (C—H = 0.95 Å) and were refined with *U*_ĩso_(H) = 1.2*U*_eq_(C) using a riding model.

## Supplementary Material

Crystal structure: contains datablock(s) I. DOI: 10.1107/S2056989023000245/hb8047sup1.cif

Structure factors: contains datablock(s) I. DOI: 10.1107/S2056989023000245/hb8047Isup2.hkl

IR (top) and Raman spectra of compound 1. Given are the values for the CN stretching vibrations of the selenocyanate anion. DOI: 10.1107/S2056989023000245/hb8047sup3.jpg

IR (top) and Raman spectra of the residue obtained after the first mass loss in a TG measurement of compound 1. Given are the values for the CN stretching vibrations of the selenocyanate anion. DOI: 10.1107/S2056989023000245/hb8047sup4.jpg

Experimental PXRD pattern of the residue obtained after the first mass loss in a TG measurement of compound 1 (top) and calculated PXRD pattern for Co(NCSe)2(pyridine)2 retrieved from literature (bottom). DOI: 10.1107/S2056989023000245/hb8047sup5.jpg

CCDC reference: 2235328

Additional supporting information:  crystallographic information; 3D view; checkCIF report

## Figures and Tables

**Table 1:** Selected geometric parameters (Å, °)

Ni1—N1	2.061 (2)	Ni1—N21	2.165 (2)
Ni1—N11	2.159 (2)		
			
N1^i^—Ni1—N1	180.0	N1^i^—Ni1—N21	90.75 (9)
N1^i^—Ni1—N11	91.10 (9)	N1—Ni1—N21	89.25 (9)
N1—Ni1—N11	88.90 (9)	N11—Ni1—N21	92.40 (8)

Symmetry code: (i) 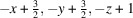 .

**Table 2:** Hydrogen-bond geometry (Å, °)

*D*—H⋯*A*	*D*—H	H⋯*A*	*D*⋯*A*	*D*—H⋯*A*
C11—H11⋯Se1^ii^	0.95	3.09	3.895 (3)	144
C11—H11⋯N1^i^	0.95	2.67	3.173 (4)	114
C12—H12⋯Se1^iii^	0.95	3.11	3.972 (3)	151
C15—H15⋯N1	0.95	2.60	3.074 (4)	111
C21—H21⋯N1	0.95	2.54	3.061 (4)	115
C22—H22⋯Se1^iv^	0.95	3.13	4.022 (3)	157
C25—H25⋯Se1^ii^	0.95	3.00	3.725 (3)	134
C25—H25⋯N1^i^	0.95	2.55	3.103 (4)	118

Symmetry codes: (i) 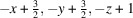 ; (ii) 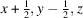 ; (iii) 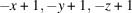 ; (iv) 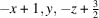 .

**Table 3:** Experimental details

Crystal data	
Chemical formula	[Ni(NCSe)_2_(C_5_H_5_N)_4_]
*M*_r_	585.07
Crystal system, space group	Monoclinic, *C*2/*c*
Temperature (K)	170
*a*, *b*, *c* (Å)	12.4422 (10), 13.2302 (9), 15.0723 (12)
β (°)	108.755 (9)
*V* (Å^3^)	2349.4 (3)
*Z*	4
Radiation type	Mo *K*α
μ (mm^−1^)	3.95
Crystal size (mm)	0.50 × 0.40 × 0.30

Data collection	
Diffractometer	Stoe *IPDS2*
Absorption correction	Numerical (*X-SHAPE* and *X-RED* 32; Stoe, 2008 ▸)
*T*_min_, *T*_max_	0.486, 0.563
No. of measured, independent and observed [*I* > 2σ(*I*)] reflections	7129, 2485, 1971
*R*_int_	0.034
(sin θ/λ)_max_ (Å^−1^)	0.639

Refinement	
*R*[*F* ^2^ > 2σ(*F* ^2^)], *wR*(*F* ^2^), *S*	0.030, 0.075, 1.02
No. of reflections	2485
No. of parameters	142
H-atom treatment	H-atom parameters constrained
Δρ_max_, Δρ_min_ (e Å^−3^)	0.89, −0.64

Computer programs: *X-AREA* (Stoe, 2008 ▸), *SHELXT2014/5* (Sheldrick, 2015*a* ▸), *SHELXL2016/6* (Sheldrick, 2015*b* ▸), *DIAMOND* (Brandenburg & Putz, 1999 ▸) and *publCIF* (Westrip, 2010 ▸).

## supplementary crystallographic information

### Crystal data

**Table:**

[Ni(NCSe)_2_(C_5_H_5_N)_4_]	*F*(000) = 1160
*M**_r_* = 585.07	*D*_x_ = 1.654 Mg m^−^^3^
Monoclinic, *C*2/*c*	Mo *K*α radiation, λ = 0.71073 Å
*a* = 12.4422 (10) Å	Cell parameters from 7129 reflections
*b* = 13.2302 (9) Å	θ = 2.3–27.0°
*c* = 15.0723 (12) Å	µ = 3.95 mm^−^^1^
β = 108.755 (9)°	*T* = 170 K
*V* = 2349.4 (3) Å^3^	Block, purple
*Z* = 4	0.50 × 0.40 × 0.30 mm

### Data collection

**Table:**

Stoe IPDS-2 diffractometer	1971 reflections with *I* > 2σ(*I*)
ω scans	*R*_int_ = 0.034
Absorption correction: numerical (X-Shape and X-Red 32; Stoe, 2008)	θ_max_ = 27.0°, θ_min_ = 2.3°
*T*_min_ = 0.486, *T*_max_ = 0.563	*h* = −15→15
7129 measured reflections	*k* = −13→16
2485 independent reflections	*l* = −19→19

### Refinement

**Table:**

Refinement on *F*^2^	Primary atom site location: dual
Least-squares matrix: full	Hydrogen site location: inferred from neighbouring sites
*R*[*F*^2^ > 2σ(*F*^2^)] = 0.030	H-atom parameters constrained
*wR*(*F*^2^) = 0.075	*w* = 1/[σ^2^(*F*_o_^2^) + (0.0461*P*)^2^] where *P* = (*F*_o_^2^ + 2*F*_c_^2^)/3
*S* = 1.02	(Δ/σ)_max_ < 0.001
2485 reflections	Δρ_max_ = 0.89 e Å^−^^3^
142 parameters	Δρ_min_ = −0.64 e Å^−^^3^
0 restraints	

### Special details

**Table:**

Geometry. All esds (except the esd in the dihedral angle between two l.s. planes) are estimated using the full covariance matrix. The cell esds are taken into account individually in the estimation of esds in distances, angles and torsion angles; correlations between esds in cell parameters are only used when they are defined by crystal symmetry. An approximate (isotropic) treatment of cell esds is used for estimating esds involving l.s. planes.

### Fractional atomic coordinates and isotropic or equivalent isotropic displacement parameters (Å^2^)

**Table:**

	*x*	*y*	*z*	*U*_iso_*/*U*_eq_	
Ni1	0.750000	0.750000	0.500000	0.01661 (12)	
N11	0.63560 (19)	0.62770 (17)	0.43798 (16)	0.0204 (5)	
C11	0.6697 (3)	0.5310 (2)	0.4492 (2)	0.0277 (6)	
H11	0.747512	0.517340	0.481265	0.033*	
C12	0.5971 (3)	0.4497 (3)	0.4165 (2)	0.0365 (7)	
H12	0.624361	0.382260	0.427221	0.044*	
C13	0.4841 (3)	0.4692 (3)	0.3678 (2)	0.0397 (8)	
H13	0.432159	0.415397	0.344311	0.048*	
C14	0.4487 (3)	0.5685 (3)	0.3543 (3)	0.0391 (8)	
H14	0.371805	0.584215	0.320745	0.047*	
C15	0.5265 (3)	0.6448 (2)	0.3902 (2)	0.0291 (6)	
H15	0.501079	0.712884	0.380325	0.035*	
N21	0.78775 (18)	0.68763 (17)	0.63947 (15)	0.0193 (5)	
C21	0.7547 (2)	0.7336 (2)	0.7063 (2)	0.0258 (6)	
H21	0.716401	0.796653	0.692048	0.031*	
C22	0.7742 (3)	0.6931 (3)	0.7948 (2)	0.0312 (7)	
H22	0.750093	0.728412	0.839944	0.037*	
C23	0.8288 (3)	0.6010 (3)	0.8172 (2)	0.0319 (7)	
H23	0.842008	0.571618	0.877261	0.038*	
C24	0.8638 (3)	0.5528 (2)	0.7495 (2)	0.0299 (6)	
H24	0.901305	0.489356	0.762043	0.036*	
C25	0.8428 (2)	0.5993 (2)	0.6628 (2)	0.0244 (6)	
H25	0.868951	0.566730	0.617400	0.029*	
Se1	0.41596 (3)	0.85023 (2)	0.58223 (2)	0.02991 (11)	
C1	0.5366 (2)	0.83774 (19)	0.54277 (18)	0.0196 (5)	
N1	0.6154 (2)	0.82749 (18)	0.51901 (16)	0.0228 (5)	

### Atomic displacement parameters (Å^2^)

**Table:**

	*U*^11^	*U*^22^	*U*^33^	*U*^12^	*U*^13^	*U*^23^
Ni1	0.0135 (2)	0.0166 (2)	0.0203 (2)	0.00038 (17)	0.0062 (2)	−0.00075 (18)
N11	0.0182 (12)	0.0201 (12)	0.0227 (11)	−0.0014 (9)	0.0061 (10)	−0.0024 (9)
C11	0.0268 (16)	0.0216 (15)	0.0342 (16)	0.0006 (12)	0.0090 (14)	−0.0016 (12)
C12	0.044 (2)	0.0225 (15)	0.0430 (18)	−0.0059 (14)	0.0145 (17)	−0.0052 (14)
C13	0.041 (2)	0.0353 (19)	0.0436 (19)	−0.0199 (15)	0.0143 (17)	−0.0140 (15)
C14	0.0221 (17)	0.041 (2)	0.046 (2)	−0.0095 (13)	0.0004 (16)	−0.0070 (16)
C15	0.0228 (16)	0.0275 (15)	0.0333 (16)	−0.0016 (12)	0.0038 (14)	−0.0019 (12)
N21	0.0173 (12)	0.0204 (11)	0.0201 (11)	−0.0011 (9)	0.0061 (10)	−0.0006 (9)
C21	0.0231 (15)	0.0300 (15)	0.0244 (14)	−0.0004 (11)	0.0078 (13)	−0.0056 (12)
C22	0.0315 (17)	0.0428 (18)	0.0207 (13)	−0.0025 (14)	0.0103 (14)	−0.0067 (13)
C23	0.0279 (17)	0.047 (2)	0.0198 (14)	−0.0048 (14)	0.0059 (14)	0.0037 (13)
C24	0.0295 (16)	0.0296 (16)	0.0301 (14)	0.0048 (13)	0.0088 (14)	0.0073 (13)
C25	0.0242 (16)	0.0243 (15)	0.0249 (14)	0.0029 (11)	0.0079 (13)	−0.0008 (11)
Se1	0.02946 (18)	0.02786 (17)	0.04138 (19)	0.00354 (12)	0.02390 (15)	0.00093 (13)
C1	0.0225 (14)	0.0150 (12)	0.0186 (13)	0.0024 (10)	0.0028 (12)	−0.0001 (10)
N1	0.0186 (12)	0.0235 (12)	0.0270 (12)	0.0028 (9)	0.0084 (11)	0.0007 (9)

### Geometric parameters (Å, º)

**Table:**

Ni1—N1^i^	2.061 (2)	C14—H14	0.9500
Ni1—N1	2.061 (2)	C15—H15	0.9500
Ni1—N11	2.159 (2)	N21—C25	1.342 (4)
Ni1—N11^i^	2.159 (2)	N21—C21	1.350 (3)
Ni1—N21	2.165 (2)	C21—C22	1.384 (4)
Ni1—N21^i^	2.165 (2)	C21—H21	0.9500
N11—C15	1.336 (4)	C22—C23	1.383 (5)
N11—C11	1.342 (4)	C22—H22	0.9500
C11—C12	1.388 (4)	C23—C24	1.387 (4)
C11—H11	0.9500	C23—H23	0.9500
C12—C13	1.385 (5)	C24—C25	1.391 (4)
C12—H12	0.9500	C24—H24	0.9500
C13—C14	1.380 (5)	C25—H25	0.9500
C13—H13	0.9500	Se1—C1	1.792 (3)
C14—C15	1.384 (4)	C1—N1	1.154 (3)
			
N1^i^—Ni1—N1	180.0	C13—C14—C15	119.1 (3)
N1^i^—Ni1—N11	91.10 (9)	C13—C14—H14	120.4
N1—Ni1—N11	88.90 (9)	C15—C14—H14	120.4
N1^i^—Ni1—N11^i^	88.90 (9)	N11—C15—C14	123.3 (3)
N1—Ni1—N11^i^	91.10 (9)	N11—C15—H15	118.3
N11—Ni1—N11^i^	180.00 (8)	C14—C15—H15	118.3
N1^i^—Ni1—N21	90.75 (9)	C25—N21—C21	116.7 (2)
N1—Ni1—N21	89.25 (9)	C25—N21—Ni1	121.25 (17)
N11—Ni1—N21	92.40 (8)	C21—N21—Ni1	122.01 (19)
N11^i^—Ni1—N21	87.60 (8)	N21—C21—C22	122.9 (3)
N1^i^—Ni1—N21^i^	89.25 (9)	N21—C21—H21	118.5
N1—Ni1—N21^i^	90.75 (9)	C22—C21—H21	118.5
N11—Ni1—N21^i^	87.61 (8)	C23—C22—C21	119.8 (3)
N11^i^—Ni1—N21^i^	92.40 (8)	C23—C22—H22	120.1
N21—Ni1—N21^i^	180.0	C21—C22—H22	120.1
C15—N11—C11	117.1 (3)	C22—C23—C24	118.1 (3)
C15—N11—Ni1	121.4 (2)	C22—C23—H23	120.9
C11—N11—Ni1	121.5 (2)	C24—C23—H23	120.9
N11—C11—C12	123.4 (3)	C23—C24—C25	118.7 (3)
N11—C11—H11	118.3	C23—C24—H24	120.7
C12—C11—H11	118.3	C25—C24—H24	120.7
C13—C12—C11	118.5 (3)	N21—C25—C24	123.8 (3)
C13—C12—H12	120.7	N21—C25—H25	118.1
C11—C12—H12	120.7	C24—C25—H25	118.1
C14—C13—C12	118.5 (3)	N1—C1—Se1	178.1 (2)
C14—C13—H13	120.8	C1—N1—Ni1	155.6 (2)
C12—C13—H13	120.8		

Symmetry code: (i) −*x*+3/2, −*y*+3/2, −*z*+1.

### Hydrogen-bond geometry (Å, º)

**Table:**

*D*—H···*A*	*D*—H	H···*A*	*D*···*A*	*D*—H···*A*
C11—H11···Se1^ii^	0.95	3.09	3.895 (3)	144
C11—H11···N1^i^	0.95	2.67	3.173 (4)	114
C12—H12···Se1^iii^	0.95	3.11	3.972 (3)	151
C15—H15···N1	0.95	2.60	3.074 (4)	111
C21—H21···N1	0.95	2.54	3.061 (4)	115
C22—H22···Se1^iv^	0.95	3.13	4.022 (3)	157
C25—H25···Se1^ii^	0.95	3.00	3.725 (3)	134
C25—H25···N1^i^	0.95	2.55	3.103 (4)	118

Symmetry codes: (i) −*x*+3/2, −*y*+3/2, −*z*+1; (ii) *x*+1/2, *y*−1/2, *z*; (iii) −*x*+1, −*y*+1, −*z*+1; (iv) −*x*+1, *y*, −*z*+3/2.
