# Supplementary figures and images for: Synthesis, crystal structure and properties of bis­(iso­seleno­cyanato-κN)tetra­kis­(pyridine-κN)nickel(II)
Source: Acta Crystallogr E Crystallogr Commun. 2023 Jan 12;79(Pt 2):90–4. doi: 10.1107/S2056989023000245 (PMC9912460; doi:10.1107/S2056989023000245)

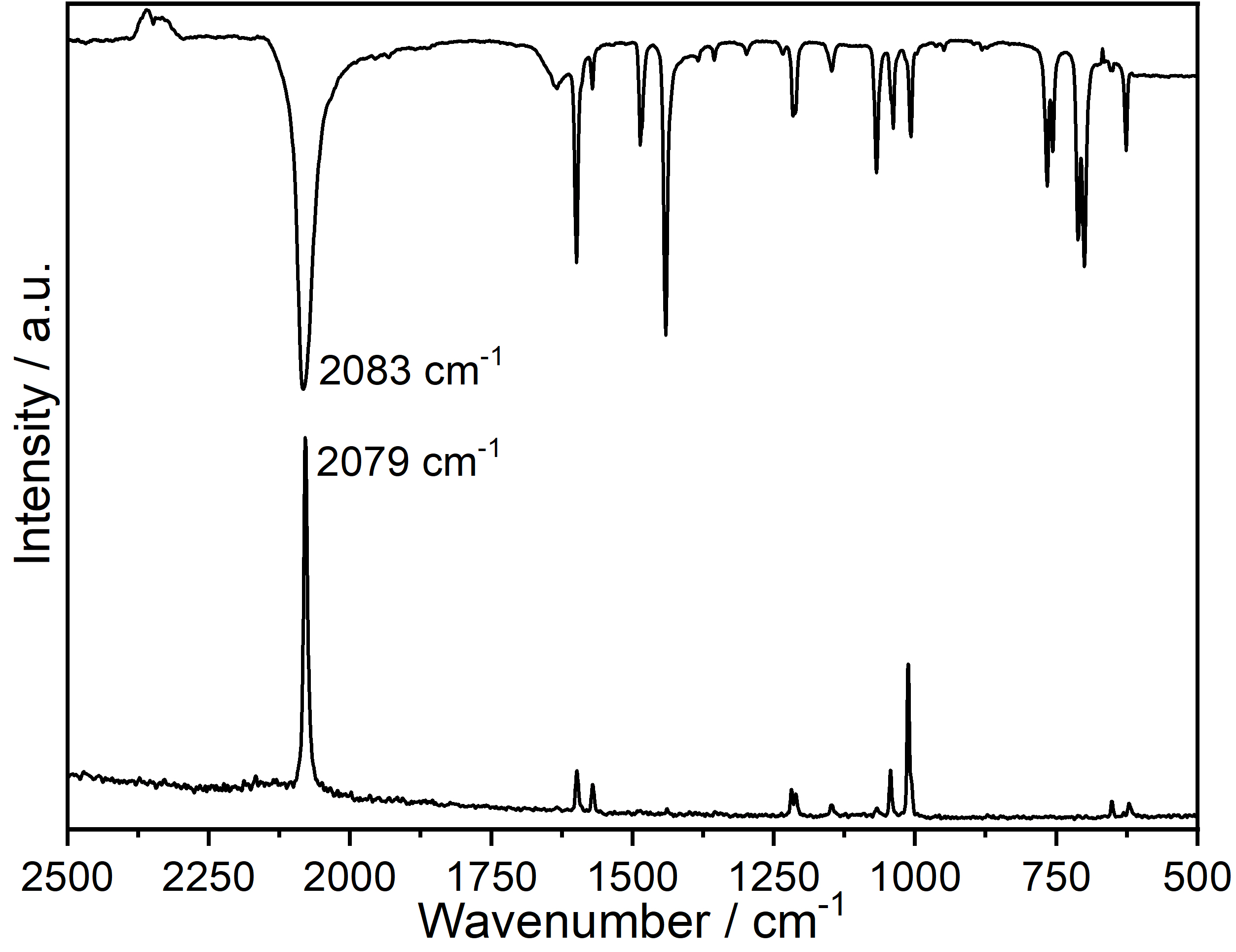

Supplement: Supplementary file 3 [file e-79-00090-sup3.jpg]

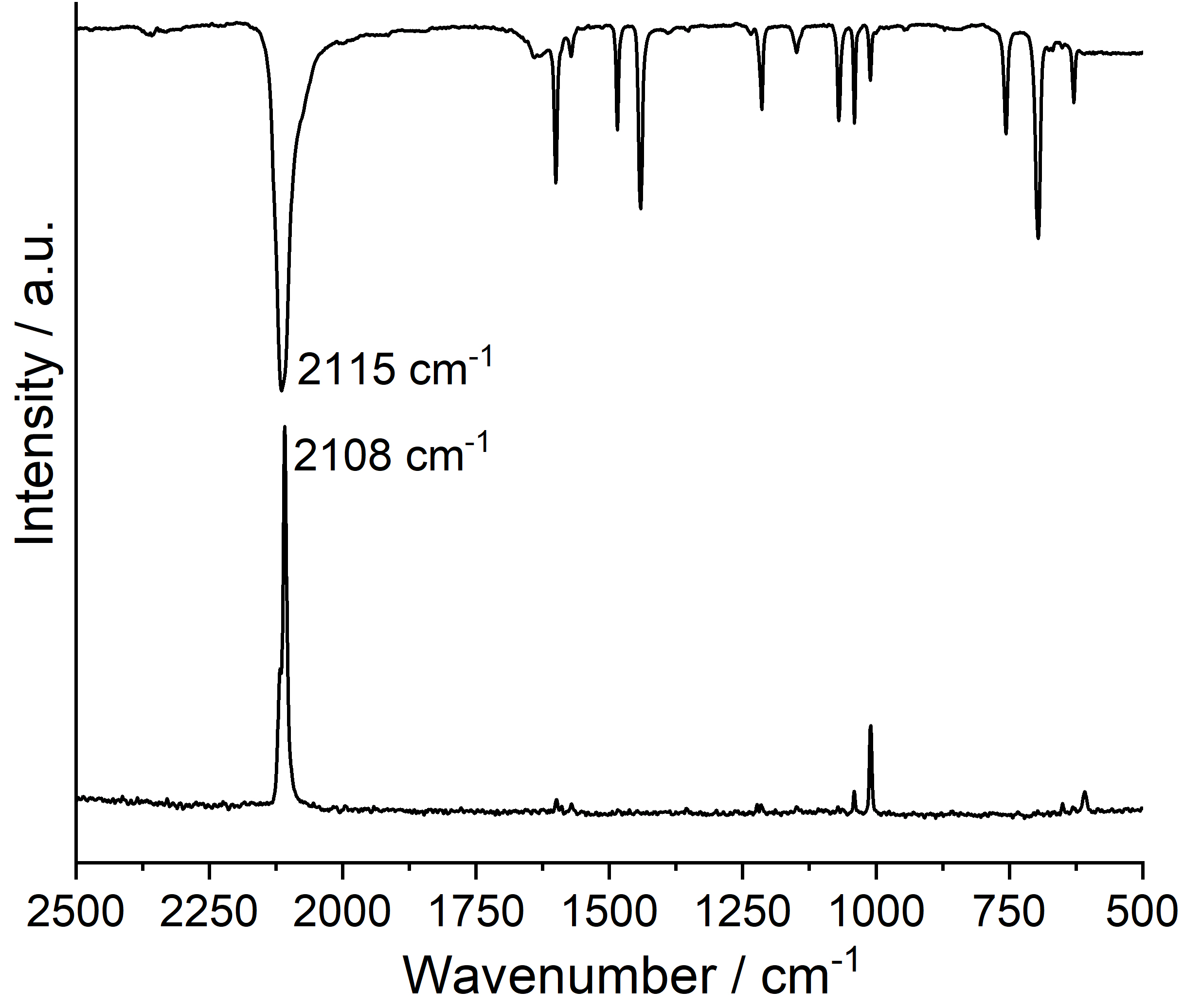

Supplement: Supplementary file 4 [file e-79-00090-sup4.jpg]

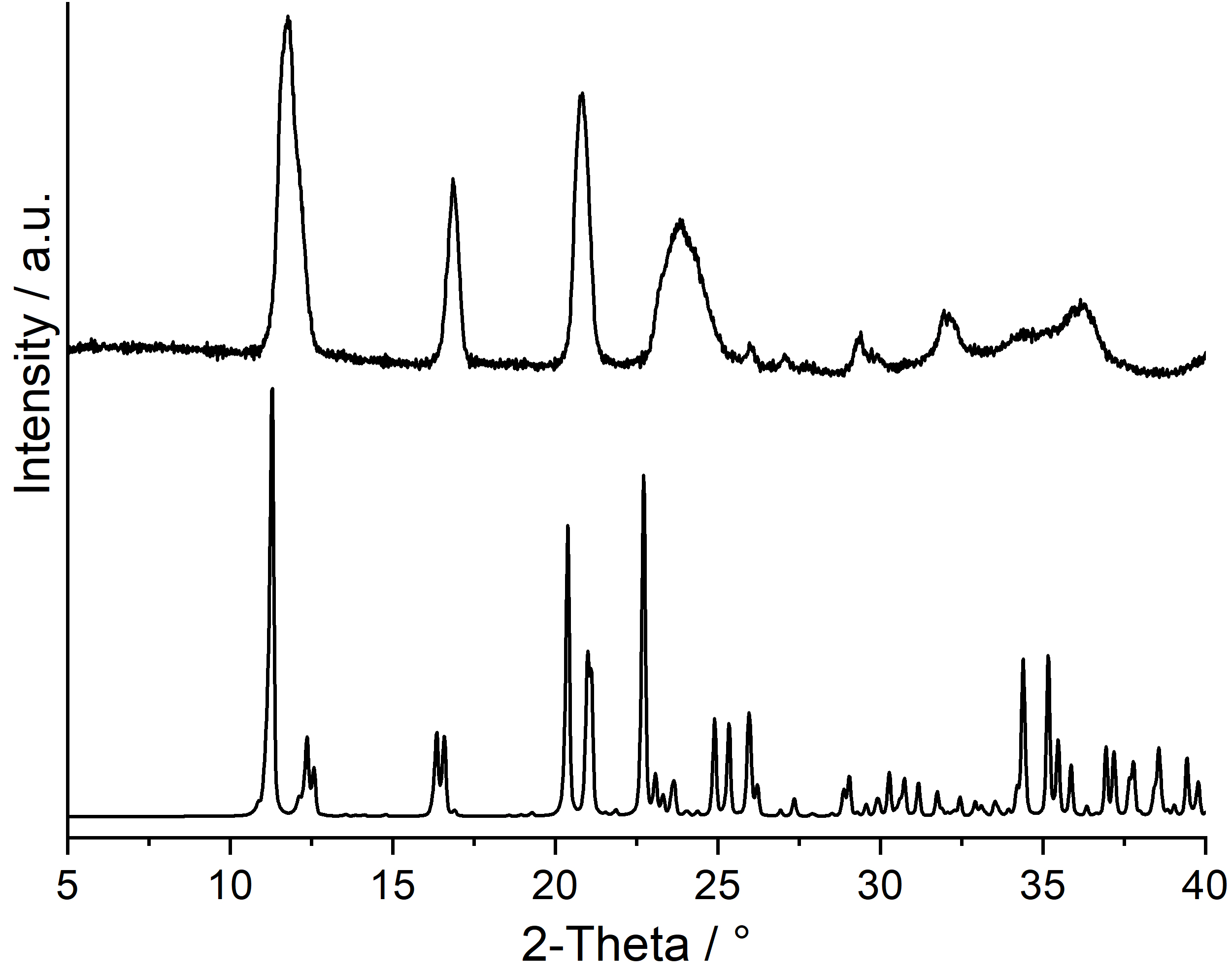

Supplement: Supplementary file 5 [file e-79-00090-sup5.jpg]
